# Improved Titer in Late-Stage Mammalian Cell Culture Manufacturing by Re-Cloning

**DOI:** 10.3390/bioengineering9040173

**Published:** 2022-04-15

**Authors:** Qin He, Matthew S. Rehmann, Jun Tian, Jianlin Xu, Luzmary Sabino, Erik Vandermark, Ziev Basson, Iris Po, Kathleen Bierilo, Gabi Tremml, Giovanni Rizzi, Erik F. Langsdorf, Nan-Xin Qian, Michael C. Borys, Anurag Khetan, Zheng-Jian Li

**Affiliations:** 1 Global Product Development and Supply, Bristol Myers Squibb Company, Devens, MA 01434, USA; matthew.s.rehmann@gmail.com (M.S.R.); juntian8@gmail.com (J.T.); luzmary.sabino@bms.com (L.S.); erik.vandermark@bms.com (E.V.); ziev@myhelaina.com (Z.B.); nanxin.qian@bms.com (N.-X.Q.); michael.borys@bms.com (M.C.B.); zli@horizontherapeutics.com (Z.-J.L.); 2 Global Product Development and Supply, Bristol Myers Squibb Company, New Brunswick, NJ 08903, USA; iris.po@bms.com (I.P.); kathleen.bierilo@bms.com (K.B.); gabi.tremml@bms.com (G.T.); giovanni.rizzi@bms.com (G.R.); erik.langsdorf@bms.com (E.F.L.); anurag.khetan@bms.com (A.K.)

**Keywords:** re-cloning, titer, biologics manufacturing, Chinese hamster ovary cells, cell culture platform

## Abstract

Improving productivity to reduce the cost of biologics manufacturing and ensure that therapeutics can reach more patients remains a major challenge faced by the biopharmaceutical industry. Chinese hamster ovary (CHO) cell lines are commonly prepared for biomanufacturing by single cell cloning post-transfection and recovery, followed by lead clone screening, generation of a research cell bank (RCB), cell culture process development, and manufacturing of a master cell bank (MCB) to be used in early phase clinical manufacturing. In this study, it was found that an additional round of cloning and clone selection from an established monoclonal RCB or MCB (i.e., re-cloning) significantly improved titer for multiple late phase monoclonal antibody upstream processes. Quality attributes remained comparable between the processes using the parental clones and the re-clones. For two CHO cells expressing different antibodies, the re-clone performance was successfully scaled up at 500-L or at 2000-L bioreactor scales, demonstrating for the first time that the re-clone is suitable for late phase and commercial manufacturing processes for improvement of titer while maintaining comparable product quality to the early phase process.

## 1. Introduction

Biologic drug demand has been increasing at an 11.9% growth rate, and sales are projected to reach USD 625.6 billion by 2026 [1]. The biopharmaceutical industry is facing a major challenge to reduce the cost of biologics manufacturing; thus ensuring that biologic drugs can reach more patients. In order to reduce manufacturing cost, manufacturing titer can be improved by increasing specific productivities (Qp) or viable cell densities (VCD) [2,3,4], often through cell line development [5,6,7] or medium and process optimization [4,8,9,10,11].

Chinese hamster ovary (CHO) cells are the most used host cells for manufacturing of biologics [12,13,14]. During CHO cell line development, the most common gene selection systems are dihydrofolate reductase (DHFR) or L-glutamine synthetase (GS) [15,16,17,18]. The GS-based system has been used in CHO cell lines that have one endogenous GS deletion or a double knockout, introducing the gene of interest along with an exogenous GS gene in the expression construct and often using medium with the selection reagent methionine sulfoximine (MSX) that inhibits GS enzymatic activity [15]. The use of the GS-based system has become increasingly common due to several advantages over other selection systems, which include (i) fewer amplification steps required to achieve higher titer, leading to reduced cell line development timelines compared to the DHFR-based system, (ii) improved selection stringency and efficiency in GS knockout cell lines with construct promoter engineering, and (iii) GS overexpression that reduces ammonium levels in the cell culture by converting glutamate and ammonium into the essential amino acid glutamine [12,15,19].

Regardless of the gene selection system, CHO cells exhibit genomic instability including mutations and changes in gene copy number [20,21,22,23], chromosome rearrangement (i.e., changes in chromosome structure and number) [24,25,26], and epigenetic changes to regulate gene expression by DNA methylation or histone modifications [22,27,28], after prolonged culture under selection pressure. Genomic instability of CHO cells has been linked to heterogeneity of cell growth, titer and product quality attributes with aged cell cultures [23,29,30] and occasionally presents a challenge for the stability of productivity and product quality attributes in biologics manufacturing processes. However, the genomic instability inherent to CHO cells also provides opportunities to generate higher producing cell lines, and we originally hypothesized that increasing the culture time (i.e., additional passages) prior to single-cell cloning could yield higher producing clones.

Clonality of the production cell line is important for monoclonal antibody (mAb) product safety and efficacy in biologics manufacturing and should be assured during clone selection in alignment with the requirements of regulatory agencies [31,32]. However, the use of multiple rounds of cloning as a strategy to improve upstream titer is less common, and more specifically, re-cloning to improve titer after an early phase manufacturing process has not yet been reported in the literature. In this manuscript, we present a new application of re-cloning leading to increased titer in late phase process development of multiple mAbs using CHO GS knockout (GS-/-) cell lines. For three cell lines, re-cloning improved mAb titer while quality attributes were comparable between the parental clones and re-clones. Furthermore, for two of the cell lines, the cell culture process using the re-clone was successfully scaled up to large-scale bioreactors (i.e., 500-L or 2000-L), demonstrating the feasibility of using this approach to generate cell lines for GMP manufacturing.

## 2. Materials and Methods

### 2.1. Cell Lines, Media, Re-Clone and Seed Expansion

Three CHO GS-/- cell lines were used for expression of 3 proprietary human mAbs (mAb1, mAb2, and mAb3) generated from the same parental CHO GS-/- cell line. The three cell lines were cloned and cultured in proprietary chemically defined seed medium without L-glutamine and with 1× MSX concentration (as 1× MSX) following standard internal procedures at Bristol Myers Squibb (BMS) for early phase development and clinical manufacturing.

For re-cloning, three of the cell lines (producing mAb1, mAb2, and mAb3) were thawed from their parental RCB or MCB vials and cultured in seed medium containing 1× MSX for one or two passages (3–4 days per passage) and then in seed medium containing 4× MSX for up to eight passages in shake flasks. The shake flasks were cultured in a humidified incubator (Climo-Shaker, Kuhner) at 36.5 °C, 5% CO_2_, and 135 rpm. Single cell sorting (SCC) was performed by FACS (BD, San Jose, CA, USA) into 96-well plates, during which 1000 cells were sorted into single wells. The cells were then cultured with seed medium containing 4× MSX in the incubator at 36.5 °C and 5% CO_2_. Cellavista images were reviewed on days −1, 0, 3, 7, and day 10 to confirm clonality. With an average clonal outgrowth efficiency of 50–60%, confluency and static day 7 titers were tested on 523–577 clones (Supplemental Figures S1A, S2A and S3A). The top 96 clones were moved forward into fed-batch studies conducted in 24 Deep Well plates, and of those, clones with the top 24 titers were considered for further evaluation. To verify the expected sequence and search for any single amino acid substitution sequence variants, peptide mapping liquid chromatography-tandem mass spectrometry was employed.

The top 15–24 re-clones (i.e., the re-clones with the highest titers) were evaluated in 50-mL TubeSpin bioreactors with BMS proprietary chemically defined basal and feed media. Cells were inoculated at 0.5 × 10^6^ cells/mL and cultured for 14 days at 36.5 °C, 5% CO_2_, and 80% humidity. Feed was added every other day, starting on day 4, at 6% of the initial culture volume. Culture glucose levels were monitored with a Bioprofile Flex (Nova Biomedical, Waltham, MA, USA) or a glucometer and added up to 8 g/L on even days. The titers were assessed on days 4, 6, 8, 10, 12, and 14 using the Octet (ForteBio, Fremont, CA, USA), and day 14 titers were confirmed with a Protein A UPLC method (Supplemental Figures S1B, S2B and S3B). RCBs were generated for the top six re-clones based on cell doubling time, production peak viable cell density (VCD), mAb titer, and cell specific productivity (Qp).

The top six re-clones were evaluated with the lead upstream process conditions for the parental clones in 50-mL TubeSpin and glass 5-L bioreactors to identify and select the lead re-clone. To evaluate the top six re-clones, cells were thawed from RCB vials and cultured in shake flasks with the seed medium containing 4× MSX prior to inoculation of N-1 seed bioreactors. The lead re-clones were selected based on cell doubling time, production culture peak VCD, titer, specific productivity, quality attributes, mAb gene and protein sequences, productivity and genetic stability, each of which was compared with their respective parental cell line.

### 2.2. N-1 Seed Cultures

The laboratory-scale N-1 cultures were run in 5-L glass vessels (Sartorius) in fed-batch mode for mAb1. The fed-batch N-1 culture was fed starting on day 2 with a BMS proprietary feed at a fixed percentage of the initial culture volume and cultured for five days. The N-1 medium had the same composition as the inoculum medium containing 4× MSX, except additional glucose was added to the N-1 medium in some cases to prevent depletion.

The laboratory-scale N-1 cultures were run in 5-L glass vessels (Sartorius) in perfusion mode for mAb2 and mAb3. For perfusion N-1 cultures, an auxiliary alternating tangential flow (ATF; Repligen) filtration device was connected to the 5-L bioreactor to continuously perfuse spent culture medium, while fresh medium was continuously added at the same rate. The perfusion rate was controlled as a function of the biocapacitance as measured by an in-line probe (Hamilton). Perfusion was started on day 1 at a rate of 0.08 nL/cell/day for mAb2 and a rate of 0.04 nL/cell/day for mAb3. Temperature was maintained at 36.5 °C, dissolved oxygen was maintained at 40%, and pH was controlled with a setpoint of 7.1–7.2 and a proprietary deadband in the N-1 cultures.

The N-1 for the pilot plant was run in a 200L Xcellerex (Cytiva) bioreactor and a working volume of 80–85 L. The N-1 in the clinical manufacturing facility was run in a 500L Xcellerex bioreactor at a working volume of 190–215 L. Agitation at large-scale was based on alignment with historical projects (i.e., based on internal experience with these bioreactors), while the air flow strategy at large-scale was set based on alignment in vessel volumes per minute with the 5-L bioreactors. The N-1 at large-scale used the same media, including the same concentrations of glucose and MSX as the 5-L bioreactors run at laboratory-scale.

### 2.3. Fed-Batch Production Cultures

Fed-batch production bioreactor runs were performed in 50-mL TubeSpin and 5-L bioreactors for up to 14 days. Basal and feed media were specified for the three mAb re-clones based on the processes developed for the parental clones. Dissolved oxygen was maintained at 40% and pH was controlled at a setpoint of pH 7.1–7.2 and a proprietary deadband using 1 M Na_2_CO_3_ and CO_2_ for 5-L bioreactors only. Temperature was initially maintained at 36.5 °C, and, in some cases, shifted to a proprietary lower temperature to align with conditions developed for the parental clones.

The production bioreactors for the pilot plant were run in a 500-L Xcellerex (Cytiva) bioreactor and an initial working volume of 290–350 L. The production bioreactors in the clinical manufacturing facility were run in a 2000-L Xcellerex bioreactor at an initial working volume of 1050–1300 L. A similar strategy was used to scale up the large-scale production bioreactors as was used to scale the N-1 bioreactors: agitation at 500-L and 2000-L scales were determined based on internal experience with these bioreactors, while the air flow strategy at 500-L scale was aligned in vessel volumes per minute with the 5-L bioreactors. Air sparge at 2000-L scale was higher than at 500-L scale (in vessel volumes per minute) to prevent build-up of CO_2_.

### 2.4. In-Process Cell Culture and Quality Attribute Assays

Cell culture broth was sampled from each bioreactor daily and analyzed for gases, cell counts, and metabolites. Offline pH, pCO_2_, and pO_2_ were measured using a Bioprofile pHOx analyzer (Nova Biomedical, Waltham, MA, USA). VCD and cell viability were measured on a Vi-CELL XR automatic cell counter (Beckman Coulter, Jersey City, NJ, USA). Glucose, glutamine, glutamate, lactate, and ammonia were measured on a Cedex Bio HT (Roche CustomBiotech, Penzburg, Germany).

For titer measurements, the cell culture broth was centrifuged at 1000× *g* for 5–10 min, and the supernatant sample was analyzed using a Protein A UPLC method. The normalized titer was quantified as the actual titer (g/L) at each time point divided by the average of the corresponding parental clone day 14 titer (g/L) for each mAb. Overall cell specific productivity (normalized weight/cell/day) was calculated based on normalized titers [4].

The supernatant samples were purified by Protein A chromatography prior to quality measurements. The methods used for in-process quality attributes were similar to those described in our previous report [33]. Charge variant species (acidic, main, and basic) were measured by imaged capillary isoelectric focusing (iCIEF) or cation exchange chromatography (CEX). N-glycan profiles were measured using a commercially available RapiFluor-MS N-Glycan kit (Waters, Milford, MA, USA). Size exclusion chromatography was used to measure high molecular weight (HMW) and low molecular weight (LMW).

### 2.5. Copy Number Analysis

Copy number analysis was performed by real time qPCR analysis [34]. Cell pellets taken from specified age seed cultures were used to extract genomic DNA. Primers specific to each mAb were used. The copy number was determined by extrapolating from each mAb molecular standard curve that was generated by normalizing to the GAPDH gene.

### 2.6. Southern Blot Analysis

Southern blot analysis was used to determine the structural and integration profiles of the target HC and LC genes. Genomic DNA isolated from the seed culture cell pellets was digested with the restriction enzymes specific for the target gene expression plasmids. The digested DNA was subjected to agarose gel electrophoresis and hybridization with the gene-specific hybridization probes [34].

## 3. Results

### 3.1. Re-Clone Process Development for mAb1 Production

mAb1 had low titer in the first-in-human (FIH) clinical manufacturing process. In an attempt to increase the titer, the MCB was re-cloned. This cell line was believed to be a good candidate for re-cloning with 4× MSX because a previous study demonstrated that 4× MSX in the seed train increased mAb1 production titer by 11% [34].

**Figure 1:**
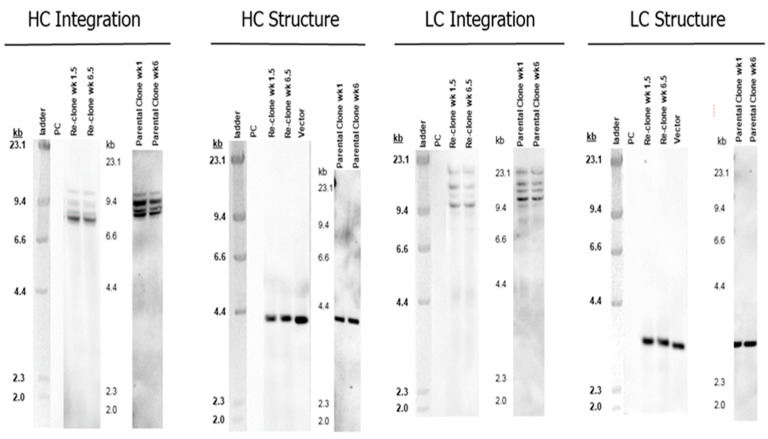
mAb1 genetic integration and structure analysis for the parental clone and re-clone. Southern blot analysis was used to determine the structural and integration profiles of the target HC and LC genes.

An MCB vial of parental mAb1 was thawed in seed media with 1× MSX, cultured for one passage and then cultured for seven passages in seed media with 4× MSX prior to single cell sorting. After single cell sorting, new RCBs were made using the re-clones. The lead re-clone and the parental clone for mAb1 had the same mAb gene sequences, the same target gene integration and structure profiles (Figure 1), and similar heavy chain and light chain gene copy numbers (GCN) (Table 1). The fed-batch titer for the re-clone increased by 62% when compared with the parental clone using the same upstream conditions in 50-mL TubeSpin bioreactors (Table 2), indicating that the re-cloning strategy increased titer beyond that obtained by just increasing MSX concentrations in seed media for mAb1.

**Figure 2:**
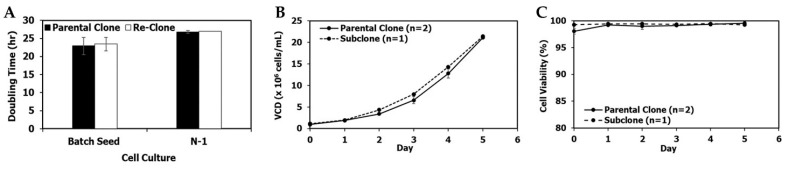
mAb1 seed and N-1 culture performance of the parental and re-clone. (**A**) Doubling times for the shake flask batch seed cultures and the N-1 5-L cultures, (**B**) N-1 fed-batch 5-L bioreactor viable cell density (VCD), (**C**) N-1 viability. The values are reported as average ± difference/2 (*n* = 2).

Figure 2A–C show a comparison between mAb1 parental clone and lead re-clone culture performance in shake flasks and N-1 fed-batch 5-L bioreactors using the same seed media containing 4× MSX and the same conditions. The average doubling time of the mAb1 re-clone was 23.4 ± 1.8 h, while the average doubling time of its parental clone was 22.9 ± 2.4 h in seven passages in shake flasks after the vial thaw (Figure 2A). The N-1 fed-batch cultures were performed in 5-L bioreactors with a target seeding density of 1.1 × 10^6^ cells/mL and a duration of 5 days. The re-clone N-1 fed-batch seed culture started at an initial viable cell density (VCD) of 1.2 × 10^6^ cells/mL and reached a final (i.e., day 5) VCD of 21.4 × 10^6^ cells/mL, leading to an average doubling time of 27.0 h. In comparison, the parental clone started at an initial VCD of 0.9 ± 0.0 × 10^6^ cells/mL and reached a final VCD of 21.1 ± 0.0 × 10^6^ cells/mL, with an average doubling time of 26.8 ± 0.34 h (Figure 2A,B). Both clones maintained high viabilities (i.e., above 95%) on day 5 (Figure 2C). Overall, seed train and N-1 cell growth for the mAb1 re-clone were comparable to the mAb1 parental clone.

**Figure 3:**
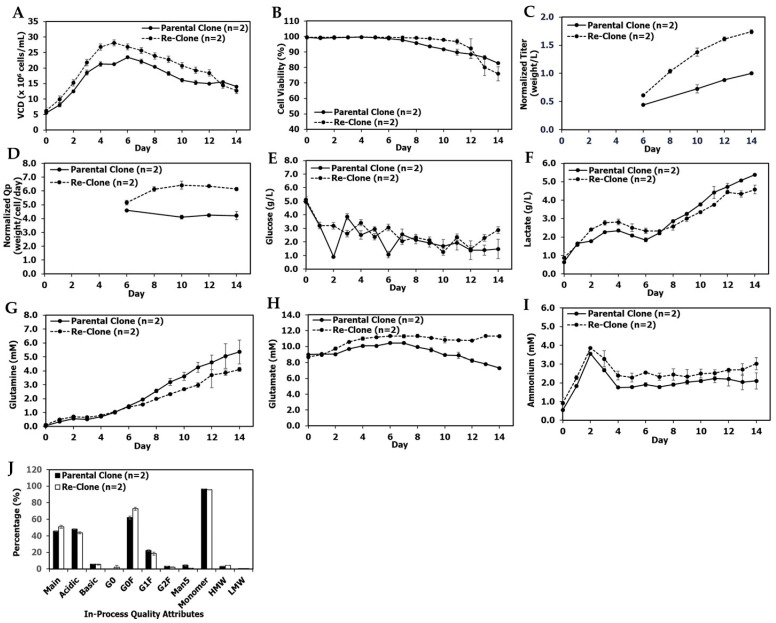
mAb1 production 5L bioreactor culture performance, metabolite and quality attribute profiles between the parental and re-clone. (**A**) Production 5-L bioreactor VCD, (**B**) production viability, (**C**) normalized titer, (**D**) normalized Qp, (**E**) production glucose profiles, (**F**) production lactate profiles, (**G**) production glutamine profiles, (**H**) production glutamate profiles, (**I**) production ammonium profiles, (**J**) in-process quality attribute profiles on day 14 at harvest. The parental clone G0 data was not available, and the day 14 titer was normalized as 1. The values are reported as average ± difference/2 (*n* = 2).

Production fed-batch cultures were performed in 5-L bioreactors with a seeding density of 6.0 × 10^6^ cells/mL and a duration of 14 days. Total feed amounts were 53% of the initial bioreactor volume, but with two minor process differences: First, feeding started on day 2 for the parental clone and on day 1 for the re-clone. Second, the parental clone was fed once per day while the re-clone was fed twice per day. Re-clone production cultures reached a peak VCD of 28.1 ± 0.1 × 10^6^ cells/mL on day 5, a final VCD of 12.7 ± 0.7 × 10^6^ cells/mL, and a final viability of 76.0% ± 4.6%; in comparison, the parental clone reached a peak VCD of 23.5 ± 0.3 × 10^6^ cells/mL on day 6, a final VCD of 14.0 ± 0.3 × 10^6^ cells/mL, and a final viability of 82.8% ± 0.6% (Figure 3A,B). The re-clone condition achieved a significantly higher day 14 normalized titer of 1.74 ± 0.04 (normalized to the day 14 titer achieved by the parental clone; Figure 3C), and a 46% higher day 14 normalized specific productivity (Qp) of 6.1 ± 0.1 weight/cell/day compared with the day 14 normalized Qp of 4.2 ± 0.2 weight/cell/day for the parental clone (Figure 3D). The re-clone cultures had similar glucose and lactate profiles, but lower glutamine levels and higher glutamate and ammonium levels towards the end of the production culture when compared with the parental clone (Figure 3E–I). The re-clone cultures had comparable quality attributes in terms of charge variants, SEC and N-glycan profiles except a higher level of G0F when compared with the parental clone (Figure 3J). These results demonstrated the mAb1 re-cloning successfully increased the titer by 74% and cell specific productivity by 46% while maintaining comparable product quality attributes (Figure 3 and Table S1).

**Figure 4:**
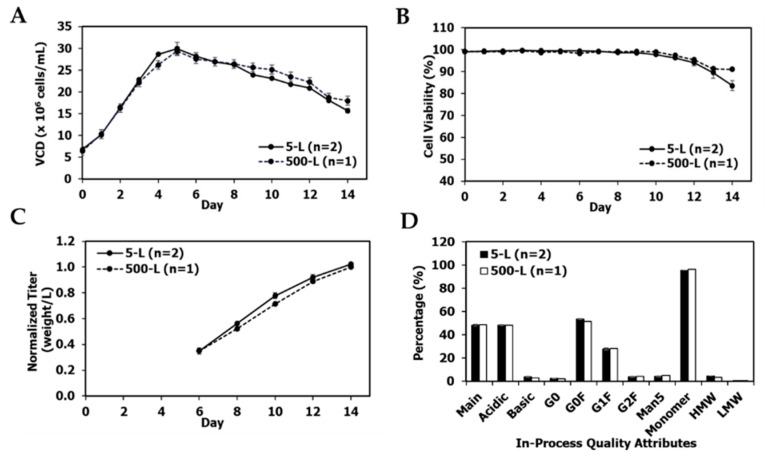
mAb1 re-clone production 500-L and 5-L satellite bioreactor culture performance, and quality attributes. (**A**) Production bioreactor VCD, (**B**) production viability, (**C**) normalized titer relative to 500-L day 14 titer, (**D**) in-process quality attribute profiles on day 14 at harvest. The values are reported as average ± difference/2 (*n* = 2).

mAb1 re-clone performance was successfully scaled up to the 500-L scale in the pilot plant (Figure 4A–D). VCD, viability, titer, and product quality were similar between the two scales.

### 3.2. Re-Clone Process Development for mAb2 Production

**Figure 5:**
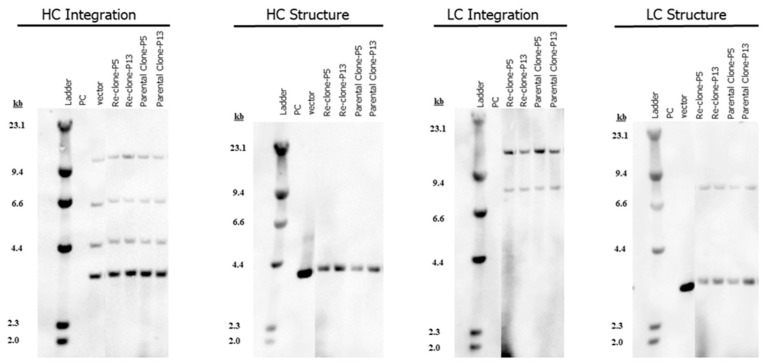
mAb2 genetic integration and structure analysis for the parental clone and re-clone. Southern blot analysis was used to determine the structural and integration profiles of the target HC and LC genes.

In order to increase productivity, re-cloning was performed for mAb2 using a similar strategy as that used for mAb1. A RCB vial of parental mAb2 was thawed in seed media with 1× MSX, cultured for one passage and then cultured for eight passages in seed media with 4× MSX before single cell sorting and RCB generation. The lead re-clone and the parental clone for mAb2 had the same mAb gene sequence, the same target gene integration and structure profiles (Figure 5), and similar HC and LC gene copy numbers at approximately the age of the working cell bank (WCB) (Table 1). Similar to mAb1, compared with the parental clone in the same conditions for mAb2, the fed-batch titer for the re-clone increased by 25% in TubeSpin bioreactors (Table 2), indicating that the re-cloning strategy also increased titer beyond that obtained by just increasing MSX concentrations in seed media for mAb2.

**Figure 6:**
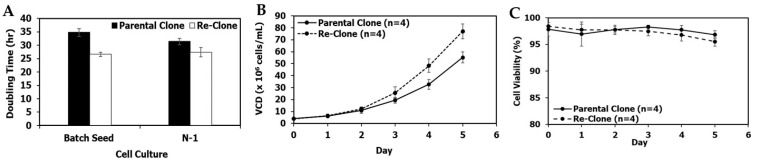
mAb2 seed and N-1 culture performance of the parental and re-clone. (**A**) Doubling times for the shake flask batch seed cultures and the N-1 5-L cultures, (**B**) N-1 fed-batch 5-L bioreactor viable cell density (VCD), (**C**) N-1 viability. The values are reported as average ± standard deviation (*n* = 4).

Figure 6A–C show a comparison between the performance of the mAb2 parental clone and lead re-clone in shake flasks and N-1 perfusion 5-L bioreactors using the same seed media and conditions. The average doubling time of the mAb2 re-clone was 26.6 ± 0.8 h, while the average doubling time of the parental clone was 34.8 ± 1.5 h in shake flasks. Thus, mAb2 re-clone doubling time was approximately 20% lower than its parental clone in seed culture prior to the N-1 culture (Figure 6A). N-1 perfusion cultures were performed in 5-L bioreactors with a target seeding density of 4 × 10^6^ cells/mL and a duration of 5 days. The perfusion seed culture for the re-clone reached a higher day 5 VCD of 77.1 ± 6.2 × 10^6^ cells/mL with a faster average doubling time of 27.4 ± 1.6 h when compared to the parental clone, which reached a day 5 VCD of 55.2 ± 4.6 × 10^6^ cells/mL with an average doubling time of 31.4 ± 1.2 h (Figure 6A,B). Both clones maintained high viabilities (i.e., above 95%) on day 5 (Figure 6C).

**Figure 7:**
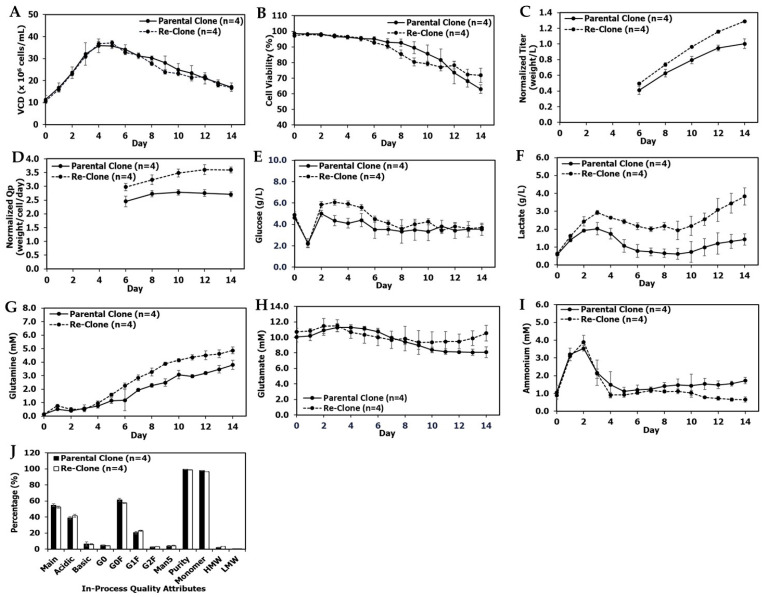
mAb2 production 5L bioreactor culture performance, metabolite and quality attribute profiles between the parental and re-clone. (**A**) Production 5-L bioreactor VCD, (**B**) production viability, (**C**) normalized titer, (**D**) normalized Qp, (**E**) production glucose profiles, (**F**) production lactate profiles, (**G**) production glutamine profiles, (**H**) production glutamate profiles, (**I**) production ammonium profiles, (**J**) in-process quality attribute profiles on day 14 at harvest. The parental clone day 14 titer was normalized as 1. The values are reported as average ± standard deviation (*n* = 4).

Production fed-batch cultures were performed in 5-L bioreactors with a target seeding density of 10 × 10^6^ cells/mL and a duration of 14 days. Total feed volume was optimized for the parental clone (45%) and for the re-clone (60%), and each clone is shown in the figures in its best representative condition. Note that the optimized condition for the parental clone (i.e., 45% feed volume) was tested for the re-clone and led to lower titer, while a higher feed volume than 45% was tested for the parental clone and led to lower titer (data not shown); therefore, the re-clone for mAb2 increased the design space in which the upstream process was able to be optimized for increased titer. Re-clone production cultures reached a peak VCD of 37.2 ± 1.6 × 10^6^ cells/mL on day 5, a final VCD of 16.8 ± 1.6 × 10^6^ cells/mL, and a final viability of 71.8% ± 4.6%. In comparison, the parental clone achieved a peak VCD of 35.8 ± 1.2 × 10^6^ cells/mL on day 4, a final VCD of 17.1 ± 2.0 × 10^6^ cells/mL, and a final viability of 63.0% ± 2.7% (Figure 7A,B). The re-clone process resulted in a significantly higher day 14 normalized titer of 1.29 ± 0.01 (normalized to the parental clone day 14 titer) and a 33% higher day 14 normalized Qp of 3.6 ± 0.11 weight/cell/day compared with the day 14 normalized Qp of 2.7 ± 0.09 weight/cell/day for the parental clone (Figure 7C,D). The re-clone cultures had similar glucose profiles (Figure 7E), higher concentrations of lactate and glutamine (Figure 7F,G), similar glutamate concentrations until late timepoints (Figure 7H), and lower concentrations of ammonium in the late stages of the production culture (Figure 7I) when compared with the metabolic profiles of the parental clone. The different metabolic profiles for mAb2 parental and re-clone were partially due to the different feed volumes. Both clones had comparable quality attributes in terms of charge variants, SEC and N-glycan profiles (Figure 7J). Thus, the mAb2 re-cloning enabled process improvements that increased both the titer and Qp by approximately 30% while maintaining comparable quality attributes when compared with the optimized process for the parental clone (Figure 7 and Table S1).

### 3.3. Re-Clone Process Development for mAb3 Production

**Figure 8:**
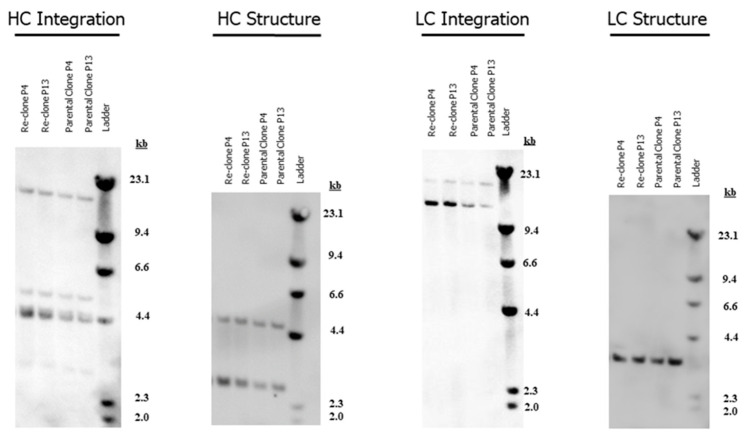
mAb3 genetic integration and structure analysis for the parental clone and re-clone. Southern blot analysis was used to determine the structural and integration profiles of the target HC and LC genes.

In order to increase mAb3 titer, a similar re-cloning strategy was applied to mAb3 during late phase process development from a RCB vial of the mAb3 parental clone. Like mAb1 and mAb2, the lead re-clone for mAb3 and the parental clone for mAb3 had the same mAb gene sequence, the same target gene integration and structure profiles (Figure 8), and similar HC and LC gene copy numbers at approximately the age of the WCB (Table 1).

**Figure 9:**
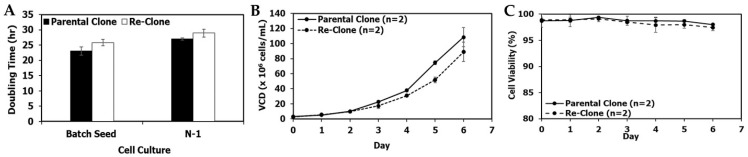
mAb3 seed and N-1 culture performance of the parental and re-clone. (**A**) Doubling times for the shake flask batch seed cultures and the N-1 5-L cultures, (**B**) N-1 fed-batch 5-L bioreactor viable cell density (VCD), (**C**) N-1 viability. The values are reported as average ± difference/2 (*n* = 2).

Compared to the parental clone with seed medium containing 1× MSX in the shake flask and N-1 seed culture, mAb3 re-clone was cultured in seed medium with 4× MSX for the seed train. The mAb3 re-clone cell growth average doubling time was 25.8 ± 1.1 h compared to the doubling time of 23.1 ± 1.4 h for the parental clone in shake flasks for the seed train (Figure 9A). The N-1 perfusion cultures were performed in 5-L bioreactors with a target seeding density of 3 × 10^6^ cells/mL and a duration of 6 days. The re-clone N-1 perfusion seed culture reached a final (i.e., day 6) VCD of 88.9 ± 13.0 × 10^6^ cells/mL with an average doubling time of 28.9 ± 1.3 h; in comparison, the parental clone achieved a day 6 VCD of 108.5 ± 12.6 × 10^6^ cells/mL with an average doubling time of 27.1 ± 0.4 h (Figure 9A,B). Both clones maintained high viabilities (i.e., above 95%) on day 6 (Figure 9C).

**Figure 10:**
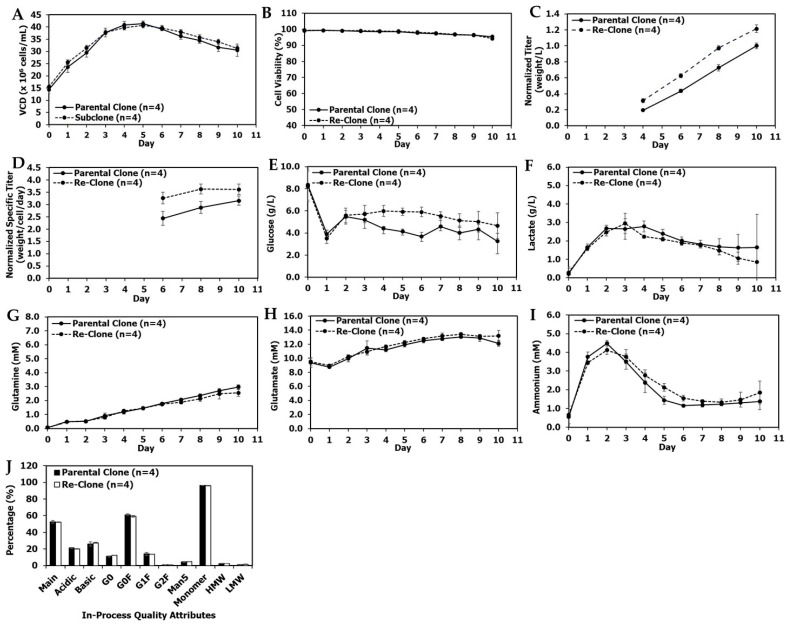
mAb3 production 5L bioreactor culture performance, metabolite and quality attribute profiles between the parental and re-clone. (**A**) Production 5-L bioreactor VCD, (**B**) production viability, (**C**) normalized titer, (**D**) normalized Qp, (**E**) production glucose profiles, (**F**) production lactate profiles, (**G**) production glutamine profiles, (**H**) production glutamate profiles, (**I**) production ammonium profiles, (**J**) in-process quality attribute profiles on day 10 at harvest. The parental clone day 10 titer was normalized as 1. The values are reported as average ± standard deviation (*n* = 4).

The production fed-batch cultures were performed at 5-L bioreactors with a target seeding density of 15 × 10^6^ cells/mL and a duration of 10 days, while other conditions were kept the same for both the parental clone and re-clone. The re-clone production culture reached a peak VCD of 40.6 ± 2.3 × 10^6^ cells/mL on day 5, a final VCD of 31.3 ± 1.5 × 10^6^ cells/mL, and a final viability of 94.2% ± 0.7% on day 10. The parental clone production culture reached a peak VCD of 41.4 ± 1.1 × 10^6^ cells/mL on day 5, a final VCD of 30.6 ± 2.5 ×10^6^ cells/mL, and a final viability of 95.4% ± 0.6% on day 10 (Figure 10A,B). The re-clone resulted in a higher day 10 normalized titer of 1.21 ± 0.05 (normalized to the parental clone day 10 titer; Figure 10C), and a 12.5% higher day 10 normalized Qp of 3.6 ± 0.2 weight/cell/day compared with the day 10 normalized Qp of 3.2 ± 0.2 weight/cell/day for the parental clone (Figure 10D). The re-clone cultures had similar glutamine, glutamate, lactate, and ammonium profiles to the parental clone; however, the glucose was typically at higher concentrations for the re-clone (Figure 10E–I). The parental and re-clone had comparable quality attributes in terms of charge variants, SEC, and N-glycan profiles (Figure 10J). These results demonstrated that mAb3 re-cloning successfully increased the productivity by 21% with comparable cell growth and quality attributes (Figure 10 and Table S1).

**Figure 11:**
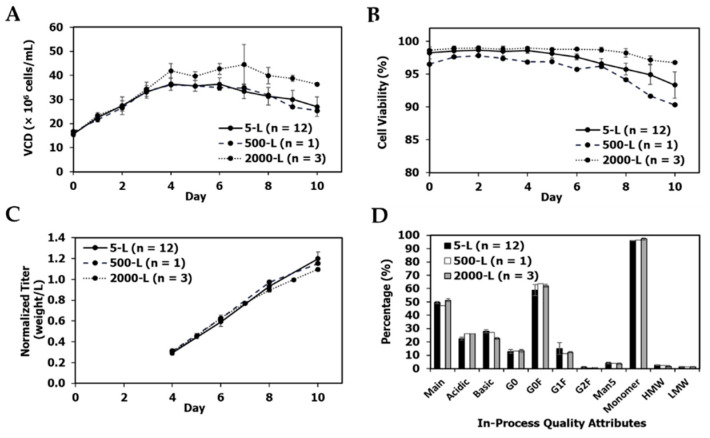
mAb3 re-clone production 500-L and 5-L satellite bioreactor culture performance, and quality attributes. (**A**) Production bioreactor VCD, (**B**) production viability, (**C**) normalized titer relative to 2000-L day 10 titer, (**D**) in-process quality attribute profiles on day 14 at harvest. Only 5-L and 2000-L values are reported as average ± standard deviation (n ≥ 3).

mAb3 re-clone performance was successfully scaled up from the 5-L bioreactor scale to the 500-L bioreactor in the pilot plant and the 2000-L bioreactor in the clinical manufacturing facility (Figure 11A–D). The process trends between 5-L and 500-L were very consistent, while the 2000-L bioreactor had slightly higher VCD and viability (Figure 11A,B). However, there was no impact from the VCD and viability differences observed at the 2000-L scale on productivity and product quality, which were similar across the three scales (Figure 11C,D).

## 4. Discussion

A primary objective of late phase or commercial upstream process development is to improve the titer of the initial process to decrease manufacturing costs without impacting product quality. Thus, the re-cloning strategy presented in this report is well-suited for late phase process development. The re-clones improved titers by 21–74% over their parental clone while maintaining similar product quality profiles (Figure 3, Figure 4, Figure 7, Figure 10 and Figure 11) for three different mAbs. To the best of our knowledge, this is the first report in the literature to demonstrate the use of re-cloning as a strategy for titer improvement, scale-up, and implementation in late phase upstream processes.

Despite differing productivities, the re-clones exhibited similar process performance to the parental cell lines, with a few exceptions, such as the effect of feed volume on the mAb2 parental clone and re-clone. Thus, the process developed for the parental clone was used as a starting point for the process development of the re-clone, and the learnings about the parental cell behavior could generally be applied to the re-clones. This demonstrates another advantage of this strategy for late phase process development: studies conducted during early phase process development on the parental clone can be leveraged for development of the re-clone process during late phase development.

The additional time investment required to implement the re-clone is approximately 2.5–3 months of cell line development. In many cases, the titer improvement is worth the additional time investment, but the decision on when and if to apply the re-cloning strategy must account for the additional resources. Importantly, the re-cloning process can be initiated in parallel to other development activities; a parallel effort ensures no or minimal impact to timelines of bringing medicines to patients. In the three studies presented here, re-cloning was initiated in parallel to upstream process development, and there was no change in project timeline from introducing the re-clone into the upstream process.

In addition to the time investment, the re-clones in this study used seed medium with a higher concentration of MSX than the parental clones. Based on our experience conducting a thorough cost-of-goods analysis for a separate project [35], the increase in cost from the increased MSX concentration in the seed train is negligible and expected to be compensated for by the increase in titer observed with these re-clones.

To evaluate the long-term stability of the re-clone banks, we assessed upstream performance for mAb2 and mAb3 after approximately 48 to 49 additional generations from WCB-aged cells and observed a small, but not practically significant titer decrease of <20% for mAb2 and mAb3 re-clone (Supplemental Figures S4 and S5). Importantly, there were no significant changes in HC and LC gene copy numbers or antibody product quality profiles after 48 to 49 additional generations from the WCB (data not shown). Based on our experience, a productivity decrease of <20% after nearly 50 generations beyond manufacturing age would not be a significant impediment in a commercial upstream process; thus, re-clones generated by the strategy applied in this report were considered sufficiently stable and suitable for late phase process development. In particular, scaling the process up to a 15,000-L bioreactor would require one to two additional passages beyond the scale-up train used for the 500-L or 2000-L processes. This would contribute approximately 3–7 additional generations to the seed train, well within the limits of what was tested here.

A review of the literature suggests two likely explanations for the increased titer produced in the processes with the re-clones. First, clonal CHO cell populations exhibit natural heterogeneity, which could enable the isolation of a re-clone with desired features (i.e., higher productivity) [20,23,29,30,36]. This heterogeneity is generally accepted in the field and, importantly, is not indicative of instability in upstream process performance or product quality [6,23,37,38]. More recently, it has been suggested that the process of single cell cloning itself can impact CHO cell epigenetics in a heritable manner, leading to altered (and possibly improved) performance [39]. The data presented in this report would be consistent with both hypotheses, and it is possible that both heterogeneity of the parental culture and epigenetic change in the re-clones play a role in the titer improvement that was observed in the re-clones.

The titer increases when using the re-cloning procedure at 4× MSX (discussed here) were greater than the titer increases from increased MSX alone (which were reported in a previous study) [34]. Specifically, mAb1 (named mAb2 in the previous report) exhibited a 62–74% titer increase with the re-clone, compared with an 11% increase through the use of higher MSX alone. Similarly, mAb2 (named mAb3 in the previous report) exhibited a 25–29% increase in titer with the re-clone, compared with a 15% increase with increased MSX concentrations alone. The previous report did not discuss mAb3 since the mAb3 parental clone did not have a consistent titer increase when cultured in increased MSX concentrations, yet the re-clone for mAb3 exhibited a titer increase of 21% over the parental clone. Thus, the re-cloning process for the three recombinant proteins discussed in this report improved upstream titer by approximately 20–60% more than the use of higher MSX alone.

In conclusion, a re-clone of the lead RCB or MCB for late phase development resulted in titer improvement of 21–74% while maintaining quality attributes for three GS-/- cell lines. Since this strategy enables an end-to-end medium (i.e., no medium base powder change) from single cell cloning up to production bioreactor at scale, there are minimal regulatory hurdles to be expected with regards to clonality, and as a result, this strategy could also be employed if late-stage cell culture media changes are anticipated. Importantly, mAb1 and mAb3 re-clone performance was scaled up to 500-L and 2000-L scale, respectively, while scale-up for mAb2 is anticipated in the future assuming positive clinical readouts. When resources allow, this re-cloning approach provides a practical and effective option to late-stage process development to improve the productivity while maintaining comparable product quality attributes in the late phase manufacturing process.

## Figures and Tables

**Table 1:** Target IgG heavy chain and light chain gene copy number comparison between the parental clone and re-clone for mAb1, mAb2, and mAb3. The values are reported as average ± standard deviation (*n* = 3).

	Clone	HC GCN	LC GCN
mAb1	Parental clone	94.9 ± 18.6	148.9 ± 23.7
	Re-clone	84.7 ± 10.7	131.0 ± 8.7
mAb2	Parental clone	40.5 ± 9.1	37.2 ± 6.8
	Re-clone	39.0 ± 1.0	35.7 ± 0.8
mAb3	Parental clone	49.4 ± 1.1	49.7 ± 0.4
	Re-clone	49.1 ± 1.7	49.6 ± 0.9

**Table 2:** Normalized day 14 titer comparison between the parental clone and re-clone in 50-mL TubeSpin bioreactors for mAb1 and mAb2 using the same media and fed-batch production conditions. The values are reported as average ± difference/2 (*n* = 2).

Cell Line	Clone	Normalized Titer
mAb1	Parental clone	1.00 ± 0.02
	Re-clone	1.62 ± 0.09
mAb2	Parental clone	1.00 ± 0.01
	Re-clone	1.25 ± 0.01

## Data Availability

Not applicable.

## Supplementary Materials

The following are available online at https://www.mdpi.com/article/10.3390/bioengineering9040173/s1, Table S1. Potency for the parental clone and re-clone normalized to the reference standard for each mAb; Figure S1. Clonal titer distribution profiles for mAb1 parental clone and re-clone screening in 96 well plates and C50 SpinTube bioreactors; Figure S2. Clonal titer distribution profiles for mAb2 parental clone and re-clone screening in 96 well plates and C50 SpinTube bioreactors; Figure S3. Clonal titer distribution profiles for mAb3 parental clone and re-clone screening in 96 well plates and C50 SpinTube bioreactors; Figure S4. Stability profiles of cell culture productivity and quality for the cell culture process using the mAb2 re-clone; Figure S5. Stability profiles of cell culture productivity and quality for the cell culture process using the mAb3 re-clone.

## References

[1] (2019). Reports and Data. Biologics Market to Reach USD 625.6 Million by 2026. GlobeNewswire.

[2] van Beers M.M., Bardor M. (2012). Minimizing Immunogenicity of Biopharmaceuticals by Controlling Critical Quality Attributes of Proteins. Biotechnol. J..

[3] Yu M., Hu Z., Pacis E., Vijayasankaran N., Shen A., Li F. (2011). Understanding the Intracellular Effect of Enhanced Nutrient Feeding toward High Titer Antibody Production Process. Biotechnol. Bioeng..

[4] Xu J., Rehmann M.S., Xu M., Zheng S., Hill C., He Q., Michael CBorys M.C., Li Z.J. (2020). Development of an Intensified Fed-Batch Production Platform with Doubled Titers Using N-1 Perfusion Seed for Cell Culture Manufacturing. Bioresour. Bioprocess..

[5] Lin P.C., Chan K.F., Kiess I.A., Tan J., Shahreel W., Wong S.Y., Song Z. (2019). Attenuated Glutamine Synthetase as a Selection Marker in Cho Cells to Efficiently Isolate Highly Productive Stable Cells for the Production of Antibodies and Other Biologics. mAbs.

[6] Wurm F.M. (2013). Cho Quasispecies—Implications for Manufacturing Processes. Processes.

[7] Ha T.K., Lee J.S., Lee G.M. (2019). Chapter 1—Platform Technology for Therapeutic Protein Production. Cell Cult. Eng..

[8] Galbraith S.C., Bhatia H., Liu H., Yoon S. (2018). Media Formulation Optimization: Current and Future Opportunities. Curr. Opin. Chem. Eng..

[9] Huang Y.M., Hu W., Rustandi E., Chang K., Yusuf-Makagiansar H., Ryll T. (2010). Maximizing Productivity of CHO Cell-Based Fed-Batch Culture Using Chemically Defined Media Conditions and Typical Manufacturing Equipment. Biotechnol. Prog..

[10] Xu J., Rehmann M.S., Xu X., Huang C., Tian J., Qian N.X., Li Z.J. (2018). Improving Titer While Maintaining Quality of Final Formulated Drug Substance Via Optimization of CHO Cell Culture Conditions in Low-Iron Chemically Defined Media. mAbs.

[11] Xu J., Rehmann M.S., Tian J., He Q., Chen J., Lee J., Borys M.C., Li Z.J. (2020). Rosmarinic Acid, a New Raw Material, Doubled Monoclonal Antibody Titer in Cell Culture Manufacturing. Biochem. Eng. J..

[12] Fan L., Frye C., Racher A. (2013). The Use of Glutamine Synthetase as a Selection Marker: Recent Advances in Chinese Hamster Ovary Cell Line Generation Processes. Pharm. Bioprocess..

[13] Chu L., Robinson D.K. (2001). Industrial Choices for Protein Production by Large-Scale Cell Culture. Curr. Opin. Biotechnol..

[14] Butler M., Spearman M. (2014). The Choice of Mammalian Cell Host and Possibilities for Glycosylation Engineering. Curr. Opin. Biotechnol..

[15] Wurm F.M. (2004). Production of Recombinant Protein Therapeutics in Cultivated Mammalian Cells. Nat. Biotechnol..

[16] Rita Costa A., Elisa Rodrigues M., Henriques M., Azeredo J., Oliveira R. (2010). Guidelines to Cell Engineering for Monoclonal Antibody Production. Eur. J. Pharm. Biopharm..

[17] Chusainow J., Yang Y.S., Yeo J.H., Toh P.C., Asvadi P., Wong N.S., Yap M.G. (2009). A Study of Monoclonal Antibody-Producing CHO Cell Lines: What Makes a Stable High Producer?. Biotechnol. Bioeng..

[18] Brown M.E., Renner G., Field R.P., Hassell T. (1992). Process Development for the Production of Recombinant Antibodies Using the Glutamine Synthetase (GS) System. Cytotechnology.

[19] Noh S.M., Shin S., Lee G.M. (2018). Comprehensive Characterization of Glutamine Synthetase-Mediated Selection for the Establishment of Recombinant CHO Cells Producing Monoclonal Antibodies. Sci. Rep..

[20] Ogata N., Nishimura A., Matsuda T., Kubota M., Omasa T. (2021). Single-Cell Transcriptome Analyses Reveal Heterogeneity in Suspension Cultures and Clonal Markers of Cho-K1 Cells. Biotechnol. Bioeng..

[21] Lewis N.E., Liu X., Li Y., Nagarajan H., Yerganian G., O’Brien E., Bordbar A., Roth A.M., Rosenbloom J., Bian C. (2013). Genomic Landscapes of Chinese Hamster Ovary Cell Lines as Revealed by the Cricetulus Griseus Draft Genome. Nat. Biotechnol..

[22] Kim M., O’Callaghan P.M., Droms K.A., James D.C. (2011). A Mechanistic Understanding of Production Instability in CHO Cell Lines Expressing Recombinant Monoclonal Antibodies. Biotechnol. Bioeng..

[23] Tharmalingam T., Barkhordarian H., Tejeda N., Daris K., Yaghmour S., Yam P., Lu F., Goudar C., Munro T., Stevens J. (2018). Characterization of Phenotypic and Genotypic Diversity in Subclones Derived from a Clonal Cell Line. Biotechnol. Prog..

[24] Lee J.S., Park J.H., Ha T.K., Samoudi M., Lewis N.E., Palsson B.O., Kildegaard H.F., Lee G.M. (2018). Revealing Key Determinants of Clonal Variation in Transgene Expression in Recombinant CHO Cells Using Targeted Genome Editing. ACS Synth. Biol..

[25] Vcelar S., VJadhav V., Melcher M., Auer N., Hrdina A., Sagmeister R., Heffner K., Puklowski A., Betenbaugh M., Wenger T. (2018). Karyotype Variation of CHO Host Cell Lines over Time in Culture Characterized by Chromosome Counting and Chromosome Painting. Biotechnol. Bioeng..

[26] Derouazi M., Martinet D., Schmutz N.B., Flaction R., Wicht M., Bertschinger M., Hacker D.L., Beckmann J.S., Wurm F.M. (2006). Genetic Characterization of CHO Production Host DG44 and Derivative Recombinant Cell Lines. Biochem. Biophys. Res. Commun..

[27] Feichtinger J., Hernández I., Fischer C., Hanscho M., Auer N., Hackl M., Jadhav V., Baumann M., Krempl P.M., Schmidl C. (2016). Comprehensive Genome and Epigenome Characterization of CHO Cells in Response to Evolutionary Pressures and over Time. Biotechnol. Bioeng..

[28] Wippermann A., Noll T. (2017). DNA Methylation in CHO Cells. J. Biotechnol..

[29] Ko P., Misaghi S., Hu Z., Zhan D., Tsukuda J., Yim M., Sanford M., Shaw D., Shiratori M., Snedecor M. (2018). Probing the Importance of Clonality: Single Cell Subcloning of Clonally Derived CHO Cell Lines Yields Widely Diverse Clones Differing in Growth, Productivity, and Product Quality. Biotechnol. Prog..

[30] Davies S.L., Lovelady C.S., Grainger R.K., Racher A.J., Young R.J., James D.C. (2013). Functional Heterogeneity and Heritability in CHO Cell Populations. Biotechnol. Bioeng..

[31] FDA (1998). Q5D Quality of Biotechnological/Biological Products: Derivation and Characterization of Cell Substrates Used for Production of Biotechnological/Biological Products.

[32] Frye C., Deshpande R., Estes S., Francissen K., Joly J., Lubiniecki A., Munro T., Russell R., Wang T., Anderson K. (2016). Industry View on the Relative Importance of “Clonality” of Biopharmaceutical-Producing Cell Lines. Biologicals.

[33] Yongky A., Xu J., Tian J., Oliveira C., Zhao J., McFarland K., Borys M.C., Li Z.J. (2019). Process Intensification in Fed-Batch Production Bioreactors Using Non-Perfusion Seed Cultures. mAbs.

[34] Tian J., He Q., Oliveira C., Qian Y., Egan S., Xu J., Qian N.X., Langsdorf E., Warrack B., Aranibar N. (2020). Increased Msx Level Improves Biological Productivity and Production Stability in Multiple Recombinant GS CHO Cell Lines. Eng. Life Sci..

[35] Xu J., Xu X., Huang C., Angelo J., Oliveira C., Xu M., Xu X., Temel D., Ding J., Ghose S. (2020). Biomanufacturing evolution from conventional to intensified processes for productivity improvement: A case study. mAbs.

[36] Huhn S., Chang M., Kumar A., Liu R., Jiang B., Betenbaugh M., Lin H., Nyberg G., Du Z. (2022). Chromosomal instability drives convergent and divergent evolution toward advantageous inherited traits in mammalian CHO bioproduction lineages. iScience.

[37] O’Callaghan P.M., Berthelot M.E., Young R.J., Graham J.W.A., Racher A.J., Aldana D. (2015). Diversity in Host Clone Performance within a Chinese Hamster Ovary Cell Line. Biotechnol. Prog..

[38] Wurm M.J., Wurm F.M. (2021). Naming CHO cells for bio-manufacturing: Genome plasticity and variant phenotypes of cell populations in bioreactors question the relevance of old names. Biotechnol. J..

[39] Weinguny M., Klanert G., Eisenhut P., Lee I., Timp W., Borth N. (2021). Subcloning Induces Changes in the DNA-Methylation Pattern of Outgrowing Chinese Hamster Ovary Cell Colonies. Biotechnol. J..

