# Supplementary material for: Improved Titer in Late-Stage Mammalian Cell Culture Manufacturing by Re-Cloning
Source: Bioengineering (Basel). 2022 Apr 15;9(4):173. doi: 10.3390/bioengineering9040173 (PMC9030702; doi:10.3390/bioengineering9040173)
Supplement: Supplementary file 1 [file bioengineering-09-00173-s001.zip › Supplementary Materials_revised.pdf]

bioengineering

Supplementary Materials:

Supplemental Table s1. Potency for the parental and re-clone normalized to the reference standard for each mAb. The re-clones passed the potency release tests, and potency values were similar between the parental clone and re-clone for mAb1 and mAb2 (parental clone data is not available for mAb3). ELISA potency assay was used for mAb1(parental clone n = 3, re-clone n = 1) and mAb3 (re-clone n = 3). A cell-based potency assay was used for mAb2 (parental clone n = 1, re-clone n = 1).

	Clone	Potency (%)
mAb1	Parental Clone	102.7 ± 10.8
	Re-clone	94.8
mAb2	Parental Clone	94.0
	Re-clone	91.8
mAb3	Parental Clone	N/A
	Re-clone	104.0 ± 2.0

Figure S1. Clonal titer distribution profiles for mAb1 parental clone and re-clone screening in 96 well plates and C50 SpinTube bioreactors S1A: Approximately 1000 single cells were plated in 96-well plates with the seed media plus 1 × MSX for the parental clone screening and 4 × MSX for the re-clone screening. The day 7 titers were normalized to the highest clonal titer of the parental or re-clone screening. S1B: Relative day 14 titers of the top clones in C50 spin tube bioreactors for the parental clone and re-clone selection. The top titer clone from the parental clones was selected for re-clone. The titers were normalized relative to the highest re-clone titer.

S1A

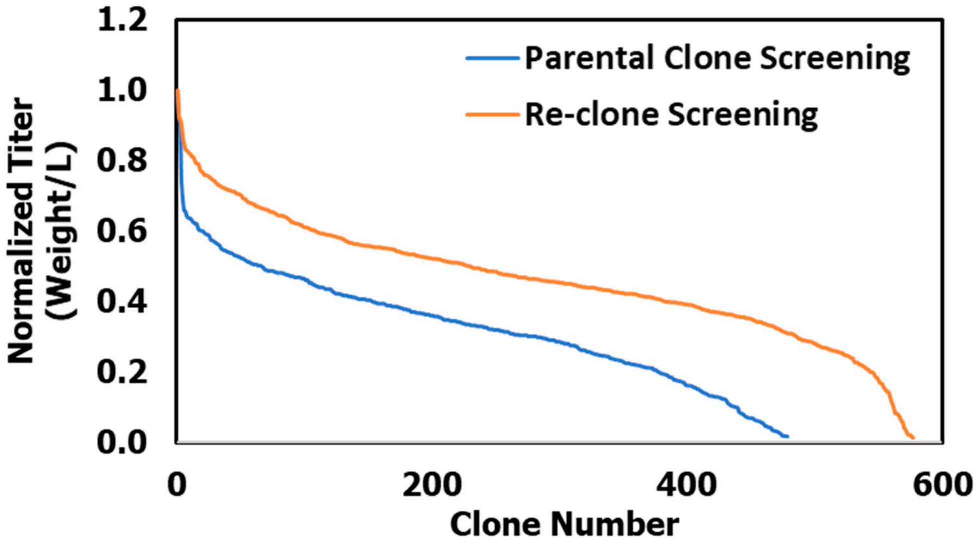

S1B

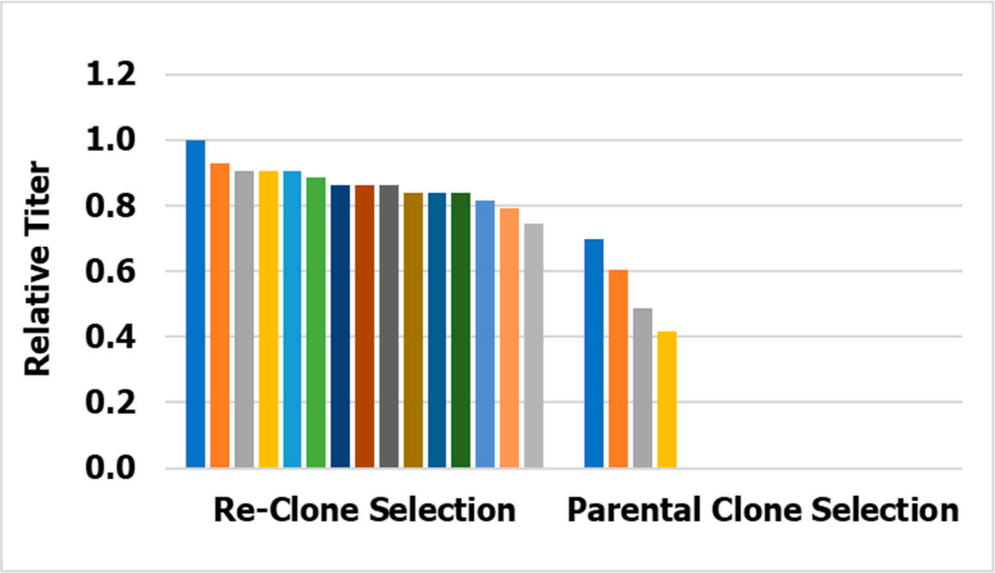

Figure S2. Clonal titer distribution profiles for mAb2 parental clone and re-clone screening in 96 well plates and C50 SpinTube bioreactors. S2A: Approximately 1000 single cells were plated in 96-well plates with the seed media plus 1 × MSX for the parental clone screening and 4 × MSX for the re-clone screening. The day 7 titers were normalized to the highest clonal titer of the parental or re-clone screening. S2B: Relative day 14

titers of the top clones in C50 bioreactors for the re-clone selection. The titers were normalized relative to the highest re-clone titer.

S2A

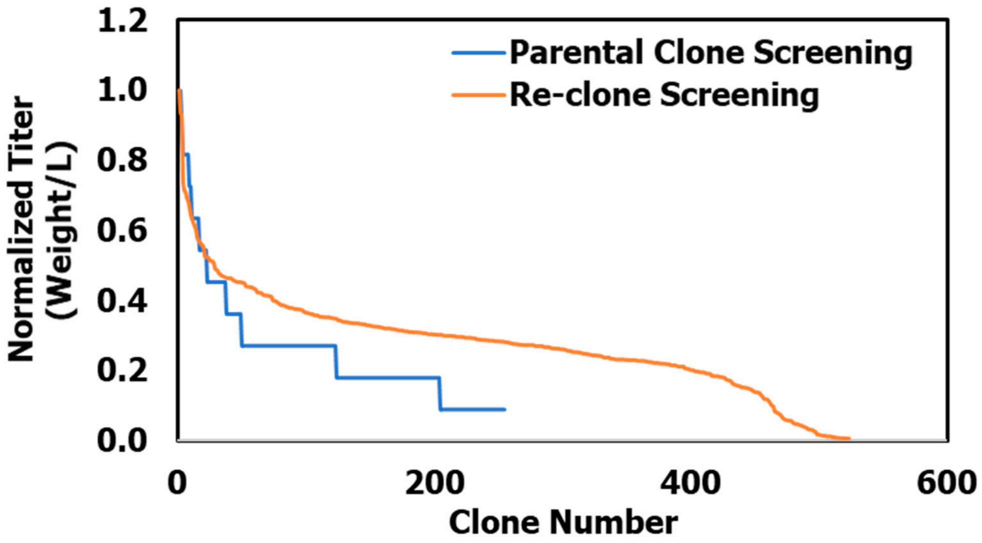

S2B

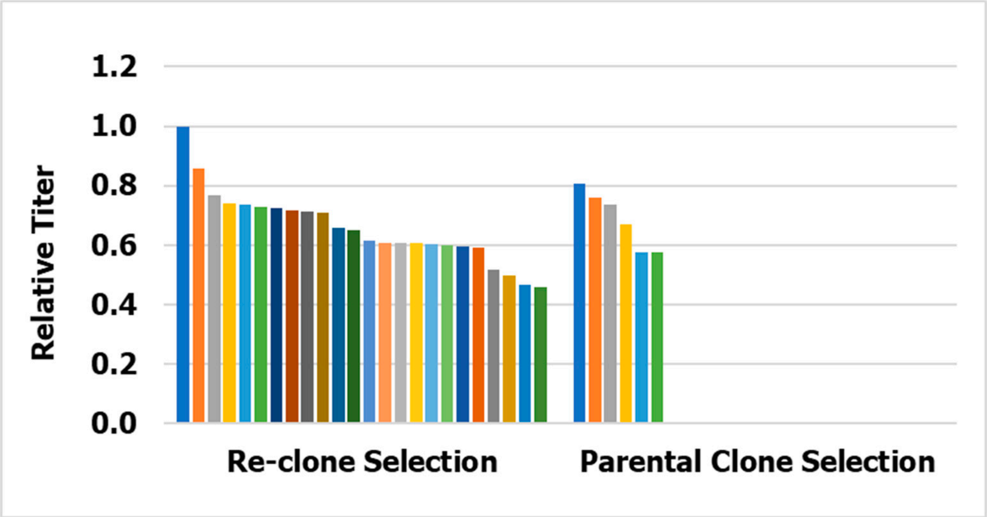

Figure S3. Clonal titer distribution profiles for mAb3 parental clone and re-clone screening in 96 well plates and C50 SpinTube bioreactors. S3A: Approximately 1000 single cells were plated in 96-well plates with the seed media plus 1 × MSX for the parental clone screening and 4 × MSX for the re-clone screening. The day 7 titers were normalized to the highest clonal titer of the parental or re-clone screening. S3B: Relative day 14 titers of the top clones in C50 bioreactors for the parental and re-clone selection. The titers were normalized relative to the highest re-clone titer.

S3A

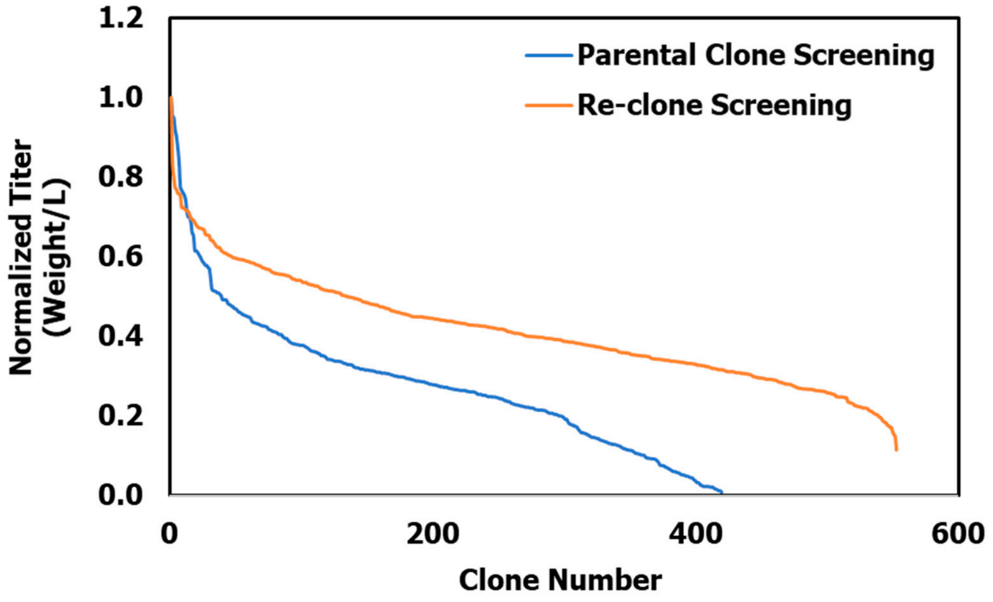

S3B

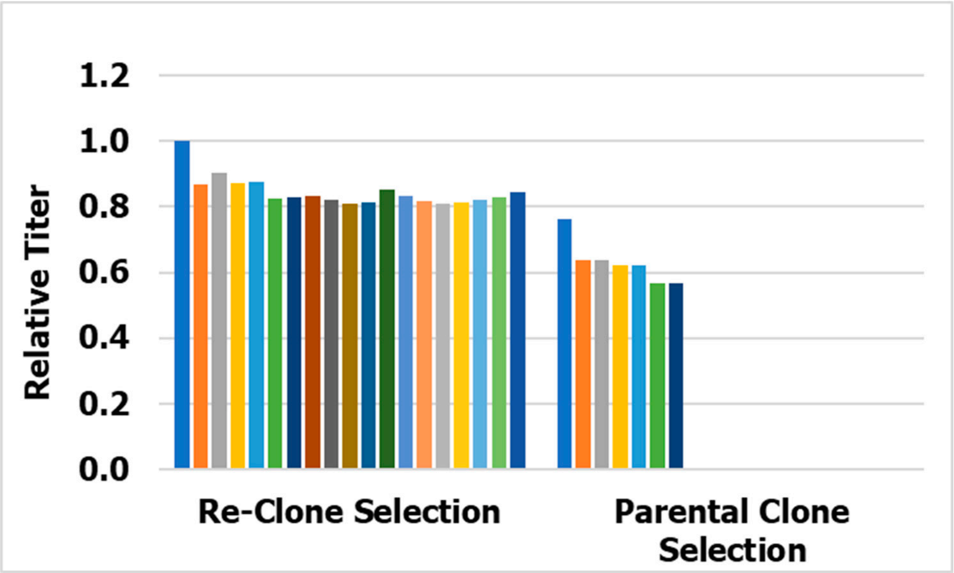

Figure S4. Stability profiles of cell culture productivity (S4A) and quality (S4B) for the cell culture process using the mAb2 re-clone at WCB-age, 30 generations after WCB-age (WCB-G30) and 48 generations after WCB-age (WCB-G48). All ages are presented as the cell age at the time of vial thaw and went through identical scale-up procedures prior to inoculation of the production bioreactor.

S4A

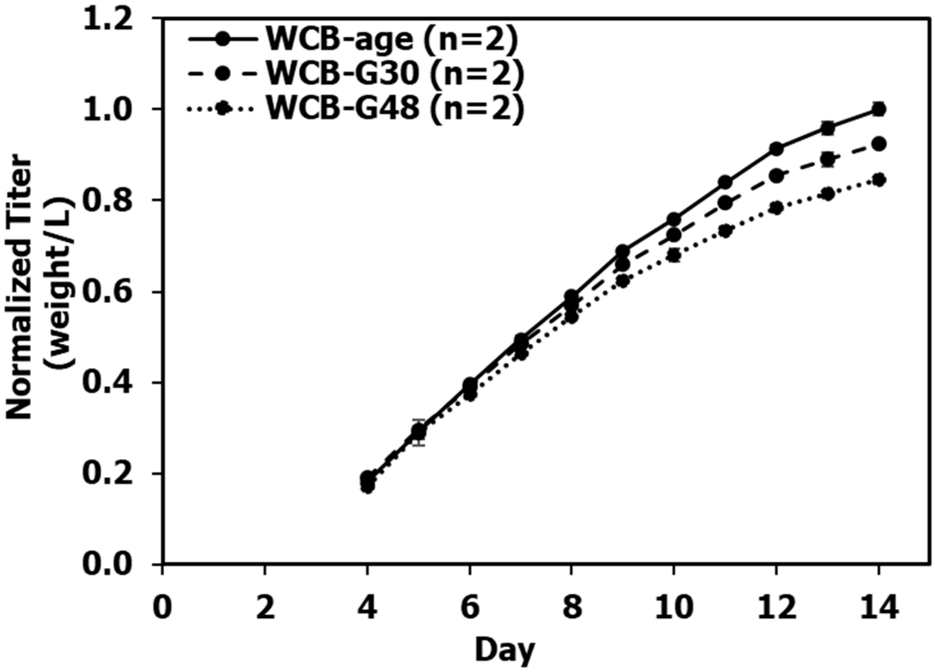

S4B

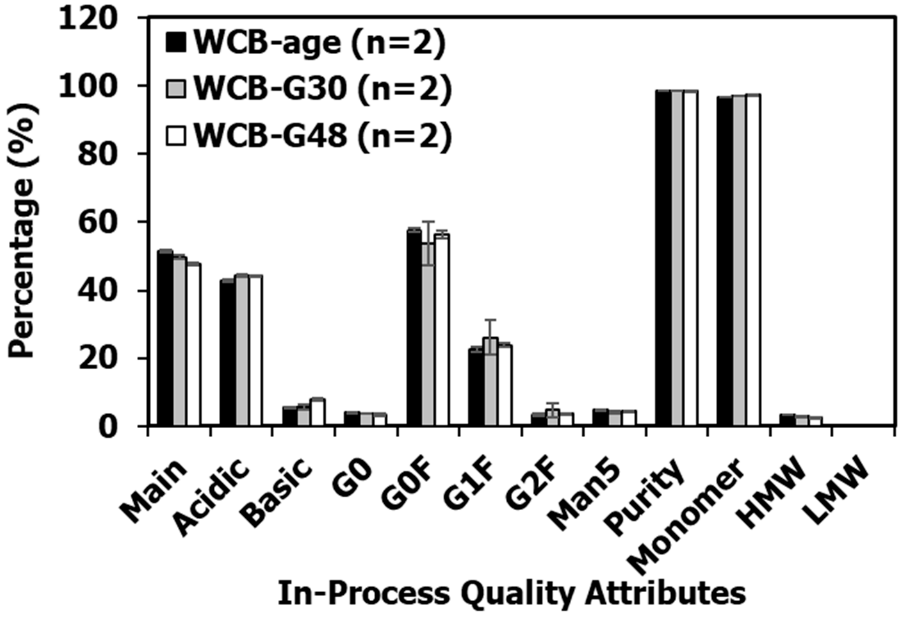

Figure S5. Stability profiles of cell culture productivity (S5A) and quality (S5B) for the cell culture process using the mAb3 re-clone at WCB-age (i.e., the same age that the cells would be after the standard scale-up train for the upstream process from the WCB), 15 generations after WCB-age (WCB-G15), 31 generations after WCB-age (WCB-G31), and 49 generations after WCB-age (WCB-G49). The stability study for mAb3 was conducted using the ambr15 high-throughput bioreactor system and titers were measured with the Cedex BioHT.

S5A

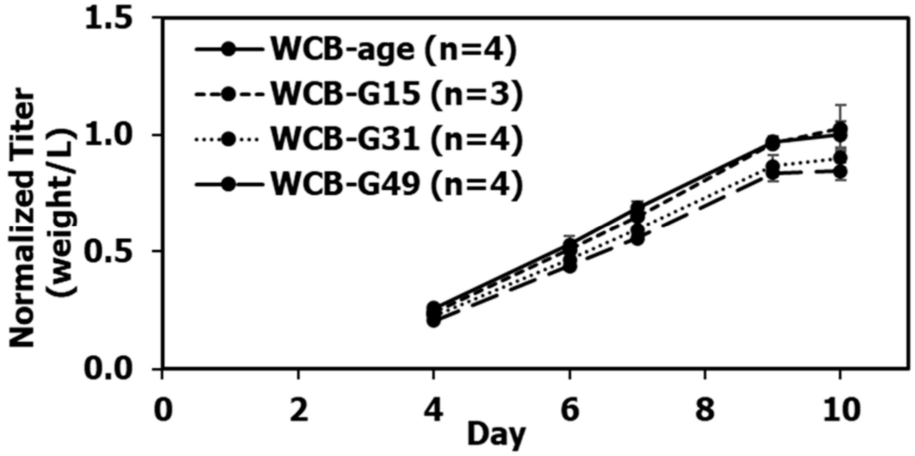
1.5
1.0
0.5
0.0
Normalized Titer (weight/L)
WCB-age (n=4)
WCB-G15 (n=3)
WCB-G31 (n=4)
WCB-G49 (n=4)
0
2
4
6
8
10
Day

S5B

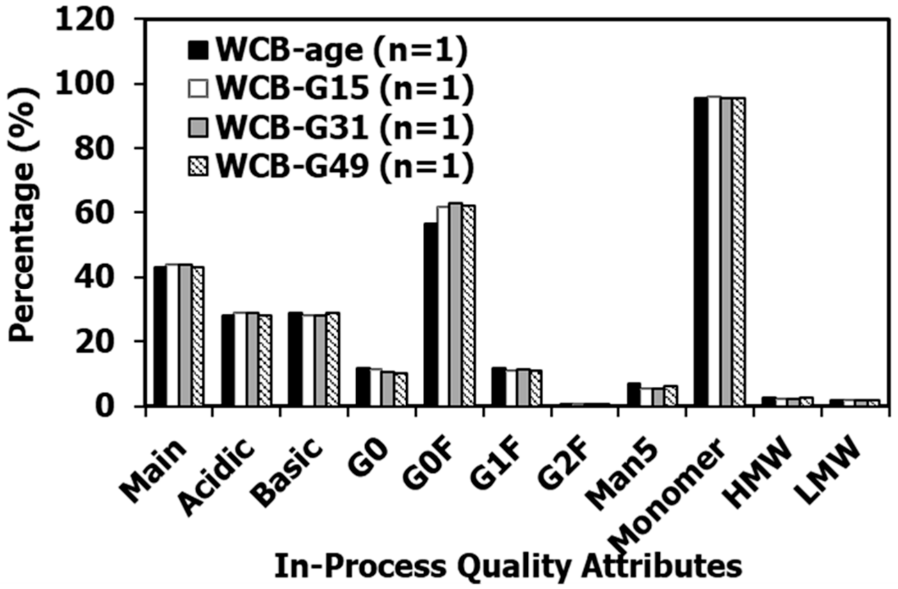
120
100
80
60
40
20
0
Percentage (%)
WCB-age (n=1)
WCB-G15 (n=1)
WCB-G31 (n=1)
WCB-G49 (n=1)
Main
Acidic
Basic
G0
G0F
G1F
G2F
Man5
Monomer
HMW
LMW
In-Process Quality Attributes
